# Clinical Characteristics of Patients with Hepatocellular Carcinoma in a Middle Eastern Population

**DOI:** 10.5812/hepatmon.7612

**Published:** 2013-05-08

**Authors:** Khalid A. Alswat, Faisal M. Sanai, Mansour Altuwaijri, Ali Albenmousa, Majid Almadi, Waleed K. Al-Hamoudi, Ayman A. Abdo

**Affiliations:** 1 Department of Medicine, Liver Disease Research Center, King Saud University, Riyadh, Saudi Arabia; 2 Department of Hepatobiliary Science and Liver Transplantation, King Abdulaziz Medical City, National Guard Health Affairs, Riyadh, Saudi Arabia; 3 Department of Gastroenterology, Riyadh Military Hospital, Riyadh, Saudi Arabia

**Keywords:** Carcinoma, Hepatocellular, Alpha-Fetoproteins, Hepatitis B, Hepatitis C, Saudi Arabia

## Abstract

**Background:**

Hepatocellular carcinoma (HCC) is one of the leading causes of death in Saudi male patients. Local clinical and demographic data of this disease are scarce.

**Objectives:**

We sought to describe the clinical characteristics and outcomes of patients from two tertiary care centers in Saudi Arabia.

**Patients and Methods:**

Data were collected for all patients diagnosed to have hepatocellular carcinoma between June 2003 and July 2008 who had been registered in a special research database (the Saudi Observatory Liver Disease Registry (SOLID)). Data were extracted from SOLID for clinical, biochemical, radiologic parameters and outcome.

**Results:**

Data was available for 363 patients, the mean age of diagnosis was 66 years, 74% of patients were males, and Hepatitis C was the underlying cause of liver disease in 48%, while Hepatitis B in 29%. Most of the patients were diagnosed at an advanced stage, 53 % of patients had a CLIP score of 4 to 6 (advanced stage), 55% had large multi-nodular tumors and 16% had vascular invasion or extra-hepatic spread at the time of diagnosis. Most of the patients had decompensated cirrhosis; with child-pogh score B in 44% and C in 26% with presence of portal hypertension in 55%. Forty eight percent died during the study period. Predictors of poor survival in the univariate analysis were; presence of portal vein thrombosis (P = 0.03), portal hypertension (P < 0.0001), presence of ascites (P = 0.022), hepatic encephalopathy (P < 0.0001), advanced child-pough score (P < 0.0001), bilirubin > 22 (P < 0.0001) and INR > 1.2 (P = 0.02). On multivariate analysis, only the presence of portal hypertension, bilirubin > 22 and severe hepatic encephalopathy were significant with adjusted hazard ratio of 1.6 (95% CI; 1.04-2.47), 1.76 (95% CI; 1.12-2.8), and 3.18 (95% CI; 1.42-7.14) respectively.

**Conclusions:**

The data from this cohort indicates that most of patients diagnosed with HCC present at late tumor and liver disease stages, when prognosis is usually dismal. Regular cancer surveillance in cirrhotic patients might change the outcomes. Further studies with results of treatment outcomes in this community are needed.

## 1. Background

Hepatocellular carcinoma (HCC) is the fifth most common cancer in men and the seventh in women (1). Its incidence differs greatly globally depending on the geographical region, ethnic group and gender. While regions like Northern Europe, North America and India have a low incidence of HCC, the opposite is true for the East, Southeast Asia and sub-Saharan Africa. Furthermore, men are three times more likely to develop HCC compared to women, independent of race and geography (1-3). In Saudi Arabia, and according to the National Cancer Registry, HCC is ranked the sixth most common cancer in males and thirteenth in females with a male to female ratio of 2.6:1. The overall age-standardized rate (ASR) is 3.5/100,000. ASR is 4.9/100,000 for males and 1.8/100,000 for females. The median age of diagnosis is 65 years for males and 60 years for females (4). These numbers are likely to significantly underestimate the true incidence of this disease, since until recently, the national registry used to require a tissue biopsy for declaring a diagnosis of HCC, which is not required for the diagnosis of HCC as per current local and international practice guidelines (5). Almost 80% of HCC cases are due to underlying chronic hepatitis B and C virus infection. Hepatitis B infection (HBV) was once considered endemic in Saudi Arabia (6-11) and a universal vaccination was introduced in 1989 (12). Similarly, in the early 90’s, the prevalence of hepatitis C infection (HCV) was found to be around 1-2% in the general population (13, 14). Although more recent studies (15) demonstrated a decreased prevalence of HBV and HCV infections, there is still a large population of infected patients who are at high risk of HCC, many of which are undiagnosed. Furthermore, most of the initial viral hepatitis studies in Saudi Arabia were conducted on children who are transitioning into adulthood and based on natural history studies, it has been estimated that about 20% of these patients will probably develop cirrhosis, with an annual risk of 1-4% for developing HCC and consequently, the incidence of HCC is expected to increase dramatically in Saudi Arabia in the next 30 years (5). Few reports have compared the clinicopathologic characteristics of patients and their impact on survival with specific reference to gender, in addition to controversial studies exist on the contribution of sex differences to patient survival and prognosis (16).

## 2. Objectives

Given the large disease burden of HCC and the scarcity of local data, we undertook this study to assess the clinicopathologic characteristics of HCC and its impact on survival.

## 3. Patients and Methods

Data were collected for all patients diagnosed to have hepatocellular carcinoma between June 2003 and July 2008 who had registered in a special research database, called the Saudi Observatory Liver Disease Registry (SOLID), (www.solid-registry.com/home.html). This included all HCC patients visited King Khalid University Hospital (KKUH) and Riyadh Military Hospital (RMH), Riyadh, Saudi Arabia during this period. Terminal follow-up was determined by the time of death or censored to the time of last follow-up for the patients labeled as alive. Data were extracted from SOLID for clinical, biochemical, radiologic parameters and outcomes. Treatment outcomes were not included as both centers did not have facilities for liver transplantation, and had limited access to conventional treatment modalities at the beginning of the study period. The ethical approval for the study was obtained from the Medical Ethics Committee of both centers. Patients were recruited in the study if they were 18 years of age or older. HCC diagnosis was confirmed based on standard published criteria (17) which include the presence of hepatic lesions with typical arterial hypervascularization and washout in the early or delayed venous phase on liver computerized tomography (CT) and/or magnetic resonance imaging (MRI). All imaging studies were read by radiologists with extensive expertise in abdominal imaging. All patients underwent either CT and/or MRI of the liver, however, needle aspiration or histological sampling were obtained only in conditions when non-invasive parameters were not diagnostic. The severity of cirrhosis was assessed by Child-Pugh-Turcotte (CPT) score. In this study, for staging of HCC, we used the Cancer of the Liver Italian Program score (CLIP) (18). This system combines tumor-related features (macroscopic tumor morphology, serum alpha-fetoprotein levels, and the presence or absence of portal vein thrombosis) with an index for the severity of cirrhosis to determine a prognostic score ranging from 0 to 6. For the purpose of the study; patients with ascites were classified into three groups (no ascites, if no ascites was documented by the imaging study; mild to moderate which was equivalent to grade 1 and 2 on the International Ascites Club criteria (19); severe, which was equivalent to grade 3 on the same criteria). Diagnosis and grading of hepatic encephalopathy was based on the commonly used classification (20). Patients were labeled to have significant portal hypertension if they had varices (esophageal and/or fundal) of any grade or a platelet count of less than 88 (x109/L), if endoscopy data was not available (21). Laboratory tests at the time of presentation including serum Alfa fetoprotein (AFP), aspartate aminotransferase (AST), alanine aminotransferase (ALT), gamma glutamyl transpeptidase (GGT), alkaline phosphatase (ALP), total bilirubin (TB), albumin, platelet count, international normalized ratio (INR), hepatitis B surface antigen (HbsAg) and anti-HCV antibodies were performed using standard, commercially available assays. AFP was measured by a conventional immunoassay (Elecsys, Roche Diagnostics GmbH, Mannheim, Germany). All AFP measurements in the HCC cases were recorded prior to any therapy for HCC.

### 3.1. Statistical Analysis

All variables were checked for normality. Descriptive statistics were summarized as mean ± standard deviation (SD) or median (range) as appropriate. Fisher’s exact test or the Chi square test were used to assess group differences for categorical variables and the Student’s t-test was used to assess differences between continuous variables. Comparison of non-parametric data was performed by Mann-Whitney U test and Kruskal-Wallis test as appropriate. Univariate and multivariate, log-rank test and Cox regression analysis were used to identify variables associated with poor survival. Missing data when less than 5%, were considered to be missing completely at random. Analyses were done with Stata version 11 (Stata Corp, Texas, USA).

## 4. Results

A total of 363 patients were included in the study and their general characteristics are summarized in Table 1.

**Table 1.** General Characteristics of All Patients

	Results
**Age, mean ± SD, y**	66.1 ± 12.14
**Sex, No. (%), Male/Female**	267 (73.6)/96(26.4)
**Etiology, No. (%)**	
HBV ^a^	98 (28.7)
HCV^a^	165 (48.2)
Combined HBV + HCV	6 (1.8)
None Viral Etiology	73 (21.3)
**CPT^ a ^ Class, No. (%)**	
A	101 (29.5)
B	152 (44.4)
C	89 (26.0)
**Ascites, No. (%)**	
No	146 (40.6)
Mild-Moderate	157 (43.6)
Severe	53 (15.8)
**Hepatic Encephalopathy, No. (%)**	
None	303 (85.8)
Grade 1-2	34 (9.6)
Grade 3-4	16 (4.5)
Significant PHT	195 (54.9)
**Albumin, No. (%)**	
< 30, g/L	190 (52.3)
≥ 30, g/L	173 (47.7)
**AFP^ a ^, No. (%)**	
< 400, ng/ml	207 (64.1)
≥ 400, ng/ml	116 (35.9)
**ALT^ a ^, median (range), U/L**	59 (7-1216)
**AST^ a ^, median (range), U/L**	80.5 (11-1309)
**GGT^ a ^, median (range), U/L**	141.5 (10-1861)
**ALP^ a ^, median (range), U/L**	188 (27-1770)
**Bilirubin, median (range), µmol/L**	25 (2-500)
**Platelet Count, median (range), x10^9^/L**	158 (33-790)
**INR^ a ^, median (range)**	1.2 (0.9-4.1)

^a^Abbreviations: AFP, alpha-fetoprotein; AST, aspartate aminotransferase; ALP, alkaline phosphatase; ALT, alanine aminotransferase; CPT, Child-Turcotte-Pugh score; GGT, gamma glutamyl transpeptidase; HBV, hepatitis B virus; HCV, hepatitis C virus; INR, international normalized ratio; PHT, portal hypertension

**Figure 1.**
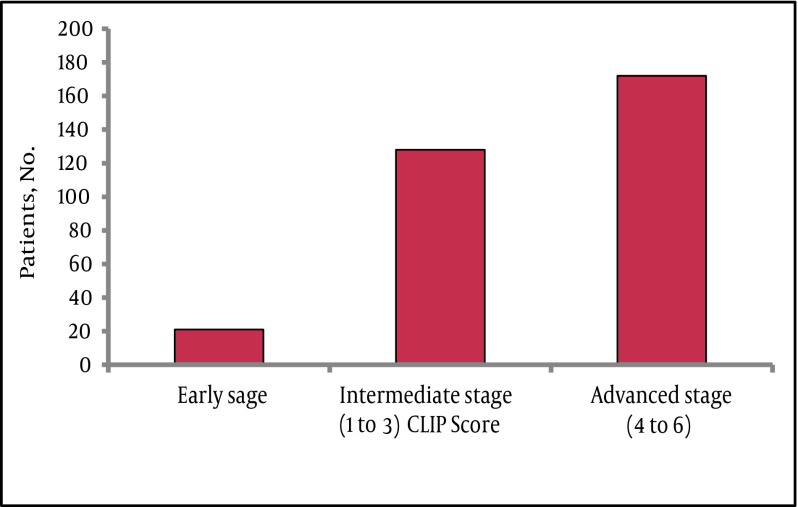
Tumor Characteristics According to the CLIP Score Abbreviations: CLIP, Cancer of the Liver Italian Program score

The mean age at time of presentation was 66.1 ± 12.1 and the majority were males 267 (73.6%) with a ratio of 3 to 1 between males and females. Statistically there was no significant difference between males and females in most of the variables, including age and liver status or tumor stage (data not shown). In this cohort HCV was the commonest underlying etiology of liver disease. Serologic markers for HCV were detected in 48.2% followed by HBV in 28.7%. Majority of patients had advanced stages of disease at the time of diagnosis, with 152 (44%) and 89 (26%) exhibiting CPT class B and C, respectively. Ascites of any degree and hepatic encephalopathy of any grade were found in 59% and 15 % of patients, respectively. Available laboratory parameters supported the presence of advanced disease in most of the patients, for example the median AST level was higher than that of ALT, 80 (11-1309) U/L vs. 59 (7- 1216) U/L respectively, also the median platelets count was158 (33-790) x109/L. Similarly, most of the patients had intermediate to advanced tumor status at presentation as assessed by the CLIP score (Figure 1).

The tumor morphology was large and multi-nodular in more than half of the patients and with vascular invasion or extra hepatic spread in 58 cases (15%). The median AFP level was 53.4 ng/ml (range 0.5-145500), with 36% of patients having an AFP more than 400 ng/ml. For each class of CPT, AFP level increased and the Pearson’s test for trend was significant (P = 0.02). AFP level did not correlate with the underlying etiology of liver disease (P = 0.20) or with the patient’s age (P = 0.59).

### 4.1. Predictors of Poor Prognosis

A total of 174 patients (48%) died during the study period. The overall median survival time was 33 months ± 5.7, (95% CI: 21.9-44.1). Survival time for males was 35 months ± 5.9, (95% CI: 23.3-46.6) and for females 18 months ± 2.7, (95% CI: 12.6-23.4).

Univariate log-rank analysis identified the following parameters to negatively influence survival: presence of portal vein thrombosis, significant portal hypertension, ascites, hepatic encephalopathy, CPT stage, bilirubin > 22 µmol/L and INR > 1.2. Other parameters such as the patient gender, CLIP score and other laboratory parameters were not significant (Table 2). Cox’s regression multivariate analysis demonstrated that only the presence of portal hypertension (adjusted hazard ratio, [aHR], 1.6; 95% CI: 1.04-2.47), bilirubin > 22 µmol/L (aHR, 1.76; 95% CI: 1.12-2.8) and advanced grade of hepatic encephalopathy (aHR, 3.18; 95% CI: 1.42-7.14) were significant (Table 3).

**Table 2.** Risks for Death by Univariate Cox Regression Analysis

	Survival Median Time, mo	Log-Rank Chi-Square Value	P value
**Sex**		3.41	0.12
Male	35		
Female	18		
**Portal vein thrombosis**		4.45	0.03
Yes	13		
No	35		
**Significant PHT^ a ^**		24.75	< 0.0001
Yes	14		
No	48		
**Ascites**		8.82	0.022
None	36		
Mild-Moderate	33		
Severe	12		
**Hepatic encephalopathy**		34.14	< 0.0001
None	37		
Grade 1-2	14		
Grade 3-4	1		
**CLIP^ a ^ score**		0.54	0.76
Early	42		
Intermediate	57		
Advanced	35		
**CPT^ a ^ class**		25.2	< 0.0001
A	68		
B	35		
C	9		
**AFP^ a ^, ng/mL**		3.17	0.075
< 400	42		
≥ 400	22		
**Age, y**		0.272	0.6
≤ 65	36		
> 65	33		
**Viral etiology of liver disease**		0.822	0.365
Yes	36		
No	22		
**ALT^ a ^, U/L**		1.73	0.19
≤ 40	24		
> 40	38		
**AST^ a ^, U/L**		0.22	0.64
≤ 37	30		
> 37	42		
**Bilirubin, µmol/L**		16.73	< 0.0001
≤ 22	48		
> 22	18		
**INR^ a ^**		5.4	0.02
≤ 1.2	42		
> 1.2	33		
**Platelet count, 10^9^/L**		0.003	0.96
≤ 150	35		
> 150	37		

^a^Abbreviations: AFP, alpha-fetoprotein; ALT, alanine aminotransferase; AST, aspartate aminotransferase; CLIP, cancer of the liver Italian program score; CPT, Child-Turcotte-Pugh score; INR, international normalized ratio; PHT, portal hypertension

**Table 3.** Multivariate Analysis of Poor Survival Predictors in Patients with HCC

	Adjusted Hazard Ratio (95% CI)
**Significant PHT ** ^a^	
Yes	1.6 (1.04, 2.47)
No	1
**Hepatic Encephalopathy**	
None	1
grade 1-2	1.27 (0.65, 2.5)
grade 3-4	3.18 (1.42, 7.14)
**Bilirubin**, µmol/L	1.76 (1.12, 2.8)
≤ 22	
> 22	1
**Portal Vein Thrombosis**	NS ^a^
Yes	
No	
**CPT^a^ class**	NS
A	
B	
C	
**Ascites**	NS
None	
mild-moderate	
Severe	
**INR** ^a^	NS
≤ 1.2	
> 1.2	

^a^Abbreviations: PHT, portal hypertension; NS, none significant; CPT, child-Turcotte-Pugh score; INR, international normalized ratio

## 5. Discussion

Hepatocellular carcinoma is an important health problem in many parts of the world, especially in areas with high viral hepatitis prevalence. In Saudi Arabia, as one of the Middle Eastern countries, HCC is considered as one of the most common health problems (4). The epidemiology of viral hepatitis in Saudi Arabia is well-described including the impact of HBV vaccination program which was introduced 2 decades ago (6-15). However, HCC as one of the serious liver disease complications is not well described. Globally, it has previously been reported that HCC patients are predominantly male and generally older (3), with a mean age of presentation between 50 and 60 years in most of the studies, however, a lower mean age of 33 years at presentation was reported in sub-Saharan Africa (22, 23). In our study, the mean age at the time of diagnosis was 66 years, which is consistent with most of the previous studies. Male patients were the majority (73.6%), with a male to female ratio of 3 to 1. This is consistent with figures from the previous Saudi Cancer Registry report, as well as other regional studies (4, 24, 25). Although previous reports suggested different median ages at diagnosis for males and females (65 and 60 years respectively), we did not find any significant difference in age between the sexes in our study population (66.7 y in men vs 64.8 in women, P = 0.18) (5). The reasons for these gender differences in the incidence and possibly disease characteristics are not well-known, yet, several factors have been suggested, such as estrogen-mediated inhibition of IL-6 production by Kupffer cells in women, which reduces both liver injury and compensatory proliferation. On the other hand, testosterone effects could increase androgen receptor signaling in men, promoting liver cell proliferation. In addition, the risk of HCC is higher in men because of the possibly of exposure to environmental liver carcinogens (such as smoking or alcohol) and higher rate of hepatitis virus infections in men (26, 27). The impact of patient’s sex on HCC characteristics’ and prognosis is controversial (28-30). We did not find significant differences between males and females in disease characteristics and prognosis. In most clinical situations, HCC develops in a cirrhotic liver (31) and, unsurprisingly, common causes of cirrhosis have been identified as key risk factors for HCC. Overall, 75% to 80% of primary liver cancers are attributed to persistent viral infection with HBV (50%-55%) or HCV (25% to 30 %). There have been numerous studies worldwide showing a strong correlation between the incidence of HCC and the prevalence of these viruses (22, 32-35). The risk factors for HCC include alcoholic liver disease and nonalcoholic fatty liver disease in addition to other less common risk factors such as hereditary hemochromatosis, alpha1-antitrypsin deficiency, autoimmune hepatitis and Wilson’s disease. The distribution of these risk factors among patients with HCC is highly variable, depending on geographic region and race (2, 36). Similar to this global figure, these viruses were the main etiologic agents of liver disease in the majority (78%) of the population we studied; HCV had a higher contributing factor (48.2%) than HBV (28.7%). Similar results were shown by Fashir et al., from a local tertiary care center, analyzing a series of 115 patients with liver masses diagnosed based on fine needle aspiration, HCC was the most common diagnosis in 87 patients (76%) with a male predominance of 82% and HBsAg and HCV antibodies were positive in 46% and 62% of patients, respectively (24). This is contradictory to several other small regional studies. For example, Fakunle et al. found 25% of their HCC patients to be anti-HCV positive and 45% positive for HBcAg (37). In the study by Ayoola and Gadour, it has been reported that HBV was more common than HCV in their population of HCC patients in the Jazan area (38). Saeed et al., also found more patients who were HBsAg positive than those who were anti-HCV positive (33.3% vs 26.2%) (39). These regional studies are small in their number of patients. In addition, some have limited geographic areas such as the study by Ayoola in the Gizan area, which has the highest HBV prevalence in the country (8). This variation of etiologic factors of HCC in our study compared to the previous regional studies could be explained by the possible impact of referral bias. Also, it is possible that the HCV predominance in our study is a result of a dramatic decline of HBV incidence, which is attributed to many factors including effective vaccination program against HBV in the last 2 decades (6, 40). Decline of HCC as a result of the vaccination program has been well-documented in Taiwan, where HCC incidence has fallen by 65 to 75% since the program began (41, 42). Since HCC generally develops in a diseased liver, the prognosis is usually affected by the status of the liver disease. Thus, most of the prognostic HCC scoring systems: like Okuda, CLIP and Barcelona included liver function impairment in the estimation of prognosis of HCC. In our study, we used the CLIP staging system to estimate prognosis of HCC in our patients as it is simple, uses common clinical criteria, and is more accurate than the Okuda, TNM and Child-Pugh staging systems. A consensus conference on staging of HCC held jointly by the American Joint Committee on Cancer (AJCC) AJCC and the American Hepatico-Pancreatico-Biliary Association in 2002 recommended that primary staging for all patients with HCC should be a clinical staging, and the CLIP system was preferred (43, 44). More recently, the Barcelona Clinic Liver Cancer (BCLC) scoring system (45) has been shown to be more clinically useful and is actually recommended by the majority of international and national authorities (46, 47). However, we were unable to describe all of its parameters adequately in our patients. Two thirds of our patients had an advanced liver disease at the time of diagnosis and the majority had an intermediate to advanced tumor stage. Liver cirrhosis was reported in 69-84% of cases in studies from Pakistan with Child’s class B or C in most of the cases (33). Our findings could be explained by the fact that both centers in this study are secondary and tertiary care centers, receiving referrals from all over the country. In addition, the possible weak implementation of international and national guidelines for surveillance of patients at risk of developing HCC resulted in a delay of diagnosis. Many observational studies (48-50) and one randomized controlled trial (51) on surveillance of HCC found that when HCC is diagnosed at an earlier stage (stage migration), survival is improved. This underscores the importance of adapting such guidelines at a national level, supervised by appropriate authorities. More recently, updated Saudi guidelines have been published and need to be disseminated and followed by all health authorities (52). Since factors related to the stage of the liver disease rather than the tumor stage were found to be most influential in patients survival, it has to be remembered that the stage of the liver disease must remain the most important factor in deciding therapeutic options and in counseling patients about HCC. Our study as a hospital-based research has some limitations with possible referral bias, as the participating centers receive cases from different regions of the country. In addition, we were unable to use the well recommended BCLC scoring system for staging as we were lacking some of its parameters. In conclusion, we have described in this study, in a large data set, the main characteristics of HCC in our population with evaluation of prognostic factors including impact of patient’s sex on disease outcomes. Our study is the largest report of HCC from this area, and it provides evidence that most of the HCC patients in Saudi Arabia present at an advanced tumor and liver disease stage, which limits the therapeutic options. This evidence is important for health authorities’ decision makers to implement some strategies in order to improve early HCC diagnosis and intervention.
